# Awareness of Bariatric Sleeve Gastrectomy Complications Among the General Population of Saudi Arabian Regions

**DOI:** 10.7759/cureus.52187

**Published:** 2024-01-12

**Authors:** Medhat Taha, Abdulaziz M Alharbi, Sara S Al-Zahrani, Hatun H Alzamzami, Bader A Alotaibi, Anmar A Alhariry, Raghad F Bahakeem

**Affiliations:** 1 Department of Anatomy, College of Medicine, Umm Al-Qura University, Al-Qunfudhah, SAU; 2 Department of Medicine, Faculty of Medicine, King Abdulaziz University, Jeddah, SAU; 3 Department of Medicine, College of Medicine, Umm Al-Qura University, Al-Qunfudhah, SAU; 4 Department of Medicine, Al-Dawadmi Medical College, Shaqra University, Riyadh, SAU; 5 Department of Medicine, King Saud Bin Abdulaziz University for Health Sciences, Jeddah, SAU; 6 Department of Medicine, College of Medicine, Umm Al-Qura University, Makkah, SAU

**Keywords:** awareness, weight loss, obesity, sleeve gastrectomy, bariatric surgery

## Abstract

Sleeve gastrectomy (SG) is a type of procedure called bariatric surgery that provides large weight loss and has a positive impact on diseases associated with obesity. However, it has brought several complications that have an impact on those undergoing surgery, which are classified into intraoperative and postoperative issues. The study's goal is to assess the Saudi Arabian population's awareness of SG consequences. This study assessed the general population's knowledge in Saudi Arabia in 2023 using a cross-sectional approach. The total number of participants was 1,013, the majority of whom were individuals between the ages of 18 and 25 (471, 46%), and females (692, 68%). A total of 692 (68%) participants showed awareness of BMI; in addition, 987 (97%) were aware of gastric sleeve surgery, and 538 (53%) understood its indications correctly. Regarding SG complications, approximately 821 (81%) of participants showed awareness. There were significant associations between knowledge of gastric sleeve surgery and residence in the northern region of Saudi Arabia. In conclusion, our study indicated that the general population is aware of the complications of gastric sleeve surgery, but it found a deficiency in their knowledge about BMI.

## Introduction

Obesity is still a major public health issue around the world, and the number of individuals affected by it is increasing. It is characterized by a BMI of 30 kg/m^2^ or more and can affect people of all ages [1]. Obesity is classified into three categories: those having a BMI of 30 kg/m^2^ to less than 35 kg/m^2^ are classified as Class 1, those having a BMI of 35 kg/m^2^ to under 40 kg/m^2^ are classified as Class 2, and those having a BMI of over 40 kg/m^2^ are classified as Class 3 [2]. Based on the WHO report in Saudi Arabia in 2019, 38% of the total population was overweight and 20% were obese, indicating that obesity is spreading at a worrying pace [3]. Obesity has been linked to various diseases, such as metabolic disorders, endocrine disorders, and joint disorders [4]. It is also strongly linked to high blood pressure, heart disease, and obstructive sleep apnea [5, 6]. The management of obesity is a step-by-step process. Obese patients have to be counseled for lifestyle modification first, which includes diet, increased physical activity, and behavior therapies. Treatment with pharmacological agents is considered the second step [7]. Pharmacological therapies for weight-loss maintenance are fairly limited. They are only approved for use in individuals with a BMI of ≥30 kg/m^2^ and those with a BMI of 27 kg/m^2^. Few drugs are used for managing obesity, one of them being orlistat, which is an irreversible pancreatic lipase inhibitor and comes with gastrointestinal side effects such as oily stool, fecal urgency, fecal incontinence, and flatulence with discharge. Liraglutide is a glucagon-like peptide 1 (GLP-1) analog, which is an incretin hormone released from the intestines in response to meals. It slows down gastrointestinal transit, enhances endogenous secretion of insulin in response to meals, alters glucose homeostasis, and suppresses appetite [2]. Surgical management is considered the most effective treatment for individuals with severe obesity, moderate obesity with other comorbidities, or patients who are not responsive to the first two steps [7].

The most popular bariatric surgery nowadays is sleeve gastrectomy (SG), which has been carried out all over the world. Sleeve gastrectomy alone has grown favorable among doctors as well as patients over the past 20 years because it is successful at reducing body mass and is technically less difficult than other traditional bariatric surgeries [8]. However, there are unfavorable complications, including stomach fistulas, hemorrhaging, leaking, and issues during the operation [9]. The early challenges are that the period following surgery is prone to a variety of issues. The most common issues patients are currently experiencing include hemorrhage, lung infections, the formation of abscesses, and infections of the wound. Furthermore, dumping usually occurs sixty minutes following meals [9, 10]. Gastroesophageal reflux disease (GERD) and stricture are both later complications [10]. Because GERD will worsen following surgery, it is one of the relative contraindications [9, 11]. Also, deficiencies in nutrition, such as thiamine deficiency (Wernicke-Korsakoff syndrome), might occur. By limiting the capacity of the stomach, the potential to absorb thiamine is significantly decreased [9, 12].

This medical study aims to determine the level of awareness among the general population in Saudi Arabia regarding SG complications and design a cross-sectional survey to collect data related to this topic. Despite the significant benefits that SG brings to patients suffering from gastric disorders, it is essential to understand and address the potential complications associated with this surgical procedure. Public awareness plays a crucial role in early detection, timely intervention, and improved patient outcomes. Currently, there is limited information available regarding the awareness of bariatric sleeve gastrectomy complications in the general population. This research seeks to fill this gap by conducting a comprehensive cross-sectional study or survey. By assessing public knowledge, perceptions, and misconceptions regarding SG complications, we can identify areas of low awareness and develop targeted educational interventions to bridge the gaps. Ultimately, improved awareness among the general population can prevent or minimize complications, empower patients to seek appropriate medical help when needed and facilitate shared decision-making between patients and healthcare providers.

## Materials and methods

This cross-sectional survey of adult Saudi citizens residing in Saudi Arabia's five regions (Central, East, West, South, and North) was carried out between July and November 2023. Both genders, aged 18 years or older, were included. The study excluded participants who had cognitive impairments or language problems. Three hundred and eighty-five was the bare minimum sample size needed to achieve. A validated Arabic/English questionnaire was used to collect the data (Appendix A). We used online distribution methods for dispersing our questionnaires at random. Our study data were collected through a pre-designed online questionnaire from a previous study conducted in the Western region of Saudi Arabia [13]. The questionnaire format is presented in Table 1. 

**Table 1:** The five-part questionnaire was divided into five sections

Section number	Details
The first section	Participants' consent form
The second section	Demographic information such as gender, level of education, age, and place of residence
The third section	Questions on general awareness and knowledge of sleeve gastrectomy
The fourth section	Assessed knowledge of sleeve gastrectomy indications
The fifth section	Assessed knowledge of complications associated with sleeve gastrectomy (95% confidence level)

Ethical approval was obtained from the Biomedical Research Ethics Committee of Umm Al-Qura University, Al-Qunfudhah, Saudi Arabia (approval number: HAPO-02-K-012-2023-09-1755). 

Statistical analysis

Data were collected in a Microsoft Excel sheet (Microsoft Corp., Redmond, WA) and analyzed using RStudio (R version 4.3.0; R Foundation for Statistical Computing, Vienna, Austria). Descriptive statistics were used, with categorical data presented as numbers and percentages. Chi-square and Fisher's exact tests were used to evaluate the determinants of knowledge of SG complications and knowledge of SG indications.

## Results

In our study, a total of 1,013 participants were enrolled, predominantly comprising individuals between 18 and 25 years of age (471, 46%), females (692, 68%), holders of bachelor's degrees (636, 63%), and residents of the Northern region (320, 32%) (Table 2).

**Table 2:** Demographic characteristics of the participants (n = 1013)

Demographic characteristics		N (%)
Age	Less than 18 years	104 (10%)
	18-25 years	471 (46%)
	26-45 years	323 (32%)
	46 years and more	115 (11%)
Gender	Female	692 (68%)
	Male	321 (32%)
Educational level	Primary	5 (0.5%)
	Intermediate	20 (2.0%)
	Secondary	309 (31%)
	Bachelor's degree	636 (63%)
	Master's degree/PhD	43 (4.2%)
Region of residence	Central	72 (7.1%)
	North	320 (32%)
	South	263 (26%)
	East	130 (13%)
	West	228 (23%)

Notably, 692 (68%) of the participants demonstrated awareness of BMI, with 35% identifying a BMI of 30 kg/m^2^ or higher as indicative of obesity. Additionally, 987 (97%) were familiar with SG, and 538 (53%) correctly understood its indications. The majority of validated Arabic and English questionnaires were used to collect the data. The majority concurred that adults with a BMI exceeding 35 kg/m^2^ and those with a BMI surpassing 40 kg/m^2^ were suitable candidates for SG (Table 3).

**Table 3:** The study group's awareness of BMI, sleeve gastrectomy, and its indications (n = 1013)

Characteristic		N (%)
Do you know what BMI is?	No	329 (32%)
	Yes	684 (68%)
What BMI qualifies a person as obese?	Less than 18.5 kg/m^2^	35 (3.5%)
	18.5-24.9 kg/m^2^	116 (11%)
	25-29.9 kg/m^2^	187 (18%)
	More than 30 kg/m^2^	357 (35%)
	I don’t know	318 (31%)
Have you heard about sleeve gastrectomy?	No	26 (2.6%)
	Yes	987 (97%)
Do you know about the indications of sleeve gastrectomy?	No	475 (47%)
	Yes	538 (53%)
What are the indications of sleeve gastrectomy?	Adults who have a BMI of less than 18.5 kg/m^2^	18 (1.8%)
	Adults with a BMI of 18.5 kg/m^2^	24 (2.4%)
	Adults with a BMI of 18.5-24.9 kg/m^2^	166 (16%)
	Adults with a BMI of more than 30 kg/m^2^ with chronic diseases	470 (46%)
	Adults with a BMI of more than 35 kg/m^2^ with chronic diseases	508 (50%)
	Adults with a BMI of more than 40 kg/m^2^	501 (49%)
	For cosmetic purposes	232 (23%)

**Figure 1:**
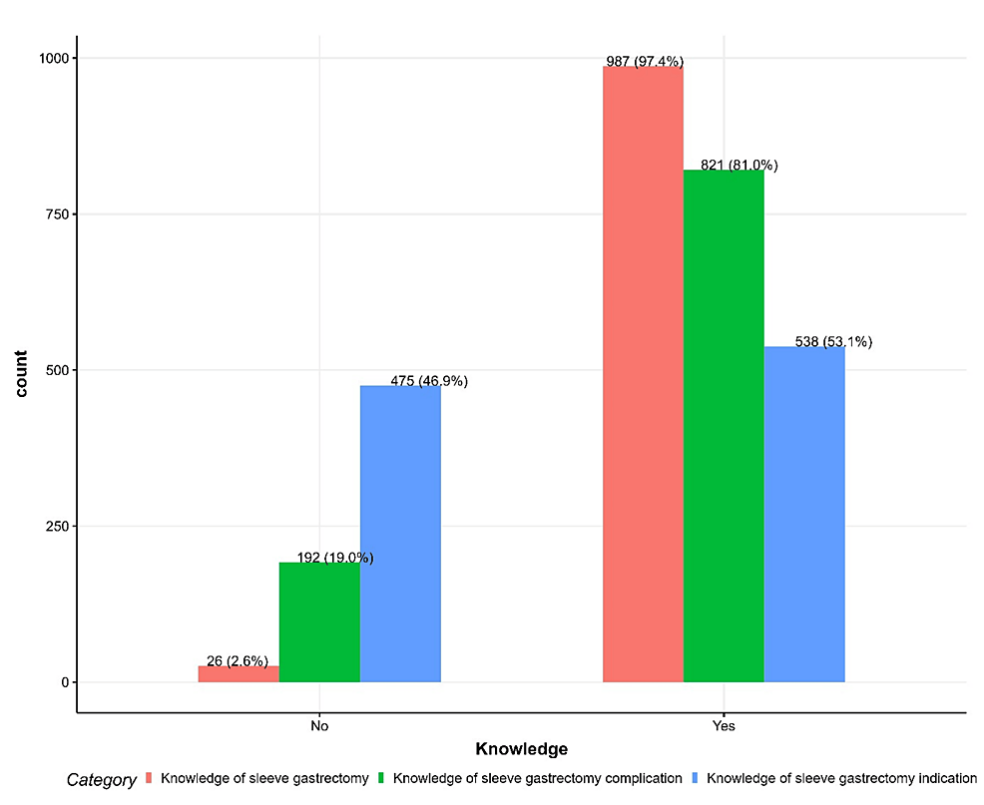
Knowledge of sleeve gastrectomy, its complications, and indications

Additionally, 988 (97.4%) were familiar with SG, and 538 (53.1%) correctly understood its indications (Figure 1).

Regarding SG complications, approximately 821 (81%) of the participants exhibited awareness. Most of the respondents were aware of hemorrhage, nutritional and mineral deficiencies, and iron deficiency as acute complications, while most of the respondents were aware of chronic complications, including anemia, iron deficiency, and nutritional and mineral deficiencies (Table 4).

**Table 4:** Association of demographic characteristics and knowledge of sleeve gastrectomy and its indication (n = 1013)

	Knowledge of sleeve gastrectomy			Knowledge of sleeve gastrectomy indications		
Characteristics	No., N = 26	Yes, N = 987	P-value	No, N = 475	Yes, N = 538	P-value
Age			0.2			0.044
Less than 18 years	6 (23%)	98 (9.9%)		42 (8.8%)	62 (12%)	
18-25 years	10 (38%)	461 (47%)		243 (51%)	228 (42%)	
26-45 years	8 (31%)	315 (32%)		144 (30%)	179 (33%)	
46 years and more	2 (7.7%)	113 (11%)		46 (9.7%)	69 (13%)	
Gender			0.5			0.083
Female	16 (62%)	676 (68%)		311 (65%)	381 (71%)	
Male	10 (38%)	311 (32%)		164 (35%)	157 (29%)	
Educational level			0.2			0.005
Primary	0 (0%)	5 (0.5%)		4 (0.8%)	1 (0.2%)	
Intermediate	0 (0%)	20 (2.0%)		13 (2.7%)	7 (1.3%)	
Secondary	4 (15%)	305 (31%)		155 (33%)	154 (29%)	
Bachelor's degree	19 (73%)	617 (63%)		292 (61%)	344 (64%)	
Master's degree/PhD	3 (12%)	40 (4.1%)		11 (2.3%)	32 (5.9%)	
Region of residence			0.031			0.024
Central	0 (0%)	72 (7.3%)		32 (6.7%)	40 (7.4%)	
North	7 (27%)	313 (32%)		159 (33%)	161 (30%)	
South	14 (54%)	249 (25%)		102 (21%)	161 (30%)	
East	1 (3.8%)	129 (13%)		62 (13%)	68 (13%)	
West	4 (15%)	224 (23%)		120 (25%)	108 (20%)	

There were significant associations between knowledge of SG and residing in the Northern region of Saudi Arabia (P = 0.031). Additionally, individuals aged 18-25 years, possessing a bachelor's degree and residing in either the Northern or Southern regions of Saudi Arabia exhibited significant knowledge of SG indications (P < 0.05) (Table 5).

**Table 5:** Awareness of the complications of sleeve gastrectomy (n=1013)

Characteristic		N (%)
Do you know the complications of sleeve gastrectomy?	No	192 (19%)
	Yes	821 (81%)
What are the acute complications of sleeve gastrectomy?	Abscess	413 (41%)
	Leaking of gastric content	393 (39%)
	Twisting of the stomach	285 (28%)
	Hemorrhage	602 (59%)
	Other nutritional and mineral deficiency	458 (45%)
	Pulmonary embolism	189 (19%)
	Weight gain	156 (15%)
	Iron deficiency	445 (44%)
	Anemia	379 (37%)
	Neuropathies	178 (18%)
What are the chronic complications of sleeve gastrectomy?	Abscess	254 (25%)
	Leaking of gastric content	308 (30%)
	Twisting of the stomach	225 (22%)
	Hemorrhage	350 (35%)
	Other nutritional and mineral deficiency	433 (43%)
	Pulmonary embolism	182 (18%)
	Weight gain	226 (22%)
	Iron deficiency	494 (49%)
	Anemia	502 (50%)
	Neuropathies	287 (28%)

Furthermore, a significant association was observed between being female, falling within the 18-25 age group, having a bachelor's education, and possessing knowledge about SG complications (P < 0.05) (Table 6).

**Table 6:** Association of demographic characteristics and sleeve gastrectomy complication knowledge (n= 1013)

	Do you know the complications of sleeve gastrectomy?		
Characteristic	No, N = 192	Yes, N = 821	P-value
Age			0.003
Less than 18 years	18 (9.4%)	86 (10%)	
18-25 years	109 (57%)	362 (44%)	
26-45 years	42 (22%)	281 (34%)	
46 years and more	23 (12%)	92 (11%)	
Gender			0.015
Female	117 (61%)	575 (70%)	
Male	75 (39%)	246 (30%)	
Educational level			0.006
Primary	0 (0%)	5 (0.6%)	
Intermediate	9 (4.7%)	11 (1.3%)	
Secondary	65 (34%)	244 (30%)	
Bachelor's degree	115 (60%)	521 (63%)	
Master's degree/PhD	3 (1.6%)	40 (4.9%)	
Region of residence			0.7
Center	11 (5.7%)	61 (7.4%)	
North	66 (34%)	254 (31%)	
South	53 (28%)	210 (26%)	
East	23 (12%)	107 (13%)	
West	39 (20%)	189 (23%)	

## Discussion

The aim of the research was to evaluate public knowledge of the indications and complications of SG. People considering bariatric surgery, including SG, should be thoroughly informed of what to anticipate. The findings of this study revealed that the knowledge of the general population was adequate. Based on our accounts, 692 (68%) were sure about the indications, and 821 (81%) knew about the complications. However, their knowledge about BMI seemed to be lacking since almost half of the subjects (475,47%) were unaware of the term BMI, and only 357 (35%) were able to identify the BMI range of obese individuals, which was 30 kg/m^2^ or more. Out of the 1,013 respondents in our poll, 692 (68%) were aware of BMI; however, only 357 (35%) defined obesity as 30 kg/m^2^. Consistent with Alolayan, research indicated that awareness about BMI was insufficient [7].

These results fall in line with the research conducted by Gowanlock et al. [14] that discovered a shortage of comprehension of SG. Of those surveyed, 41% and 64.8% had been aware of the indications and complications of the procedure, respectively, but the majority were unaware of the term BMI and had only insufficient awareness of the appropriate BMI for an obese person [15]. Of those who are unsure about the surgical indications, 475 (47%) say they are unaware, but their group's knowledge about SG and living in the Northern region of Saudi Arabia was significantly correlated (P = 0.031). Furthermore, those with a bachelor's degree who were between the ages of 18 and 25 and who lived in Saudi Arabia's Northern or Southern regions demonstrated a noteworthy level of understanding regarding the indications for sleeve gastrectomy (P < 0.05), which contradicts the findings of Alolayan's study on gender and educational attainment [7]. Moreover, 821 (81%) of the participants knew about complications, and there was a significant correlation (P < 0.05) found between the following characteristics: female gender, aged between 18 and 25, with a bachelor's degree, and aware of complications related to SG, which is consistent with the study conducted by Alamri et al. [16]. Also, the interest of females in beauty and health is an indicator of their awareness of the complications of gastric sleeve surgery [16]. Additionally, comparable with the study of Gowanlock et al. [14], the participants selected hemorrhage as the most common acute problem and anemia and iron deficiency as the most chronic complications.

A limitation of our study is that there is only one method for monitoring data, and it is preferable that more methods be considered in order to obtain better results. This was the case in some regions where the numbers were shrinking due to difficulty in reaching the participants, such as the Central region, where their number reached 70 (7.1%), followed by the Eastern region, where they reached 130 (13%), so the sample is not well distributed. Another limitation is a lack of diverse perspectives. Our result shows that 63% of participants have a bachelor’s degree, which can impact our results by being skewed towards people with higher educational backgrounds, and this can limit the representation of individuals with lower educational levels with different awareness and knowledge levels. It is now simple to find information and answers about any procedure or problem thanks to the Internet's rapid development, widespread presence, and the involvement and help of doctors on social media. We recommend that doctors post more information on social media.

## Conclusions

The study aimed to evaluate public knowledge of the indications and complications of SG. According to our study, we found that the knowledge of the general population was adequate regarding the indications and their complications; however, there was a significant lack of knowledge about BMI. Having good knowledge about BMI affects their awareness of the indications and complications of sleeve gastrectomy positively. There is still room for improvement in terms of knowledge about the subject, specifically in terms of BMI and how to correctly identify an obese person. These indicate that community awareness is necessary to increase the public's knowledge of the subject. Healthcare practitioners have the most important role in educating the community about the importance of having adequate knowledge of the indications and complications of sleeve gastrectomy.

## Appendices

Appendix A

Questionnaire

Research title: Awareness of the general population toward gastrectomy complications in Saudi Arabian regions

Research objectives 1. To assess awareness of sleeve gastrectomy complications 2. To assess awareness of sleeve gastrectomy indications

The study's goal is to measure the Saudi Arabian population's awareness of gastrectomy complications. This study examined the general population's knowledge in Saudi Arabia in 2023 using a cross-sectional approach.

This questionnaire has five sections: 1. Research description and participant consent form 2. Demographics that include age, gender, and educational level. 3. Questions assessing the general knowledge of sleeve gastrectomy (SG). 4. Questions assessing the knowledge of SG indications. 5. Questions assessing the knowledge of SG complications. After looking at each of the research titles and research objectives, answer by (✓) or (X).

First section: Consent

Would you like to participate in this questionnaire? Yes () No ()

Second section: Demographics

1. Age (in years) 18 > 18-25 25-35 35-45 45-55 55-65 > 65

2. Gender Male Female

3. Educational level Elementary school intermediate school High school Bachelor's degree Master's degree PhD

4. Region of residence Riyadh region Makkah region Madinah region Al-Bahah region Al Jawf region Al-Qassim region Asir region Eastern region Ha'il region Jizan region Najran region Northern Borders region Tabuk region

Third section: Questions assessing the general knowledge of SG

1. Have you heard about the body mass index (BMI)? Yes No

2. What is the BMI range in which we can say this person is obese? I don’t know. Under 18.5 kg/m^2^ 18.5-24.9 kg/m^2^ 25.0-29.9 kg/m^2^ 30 kg/m^2^ and above

3. Have you heard about SG? Yes No

Fourth section: questions assessing the knowledge of SG indications.

4. Do you know about the indications of SG? Yes No

5. What are the indications for SG? (choose up to 3) Adults with a BMI of more than 40 kg/m^2^ Adults with a BMI of 18.5 kg/m^2^ Adults with a BMI of 18.5-24.9 kg/m^2^ Adults with a BMI less than 18.5 kg/m^2^ Adults with a BMI greater than 35 kg/m^2^ and severe co-morbidities Adults with a BMI > 30 kg/m^2^ have poorly controlled type 2 diabetes and increased cardiovascular risk. For cosmetic reasons

Fifth section: Questions assessing the knowledge of SG complications.

6. Do you know about the complications of SG? Yes No

7. Which one of these is an acute complication of SG?? (Choose up to five options.) Hemorrhage Anemia Iron deficiency Abscess Neuropathies Weight regain A leak of gastric content Other nutritional and mineral deficiencies Pulmonary emboli Twisting of the stomach

8. Which one of these is considered a chronic complication of sleeve gastrectomy? (Choose up to five options.) Hemorrhage Anemia Iron deficiency Abscess Neuropathies Weight regain A leak of gastric content Other nutritional and mineral deficiencies Pulmonary emboli Twisting of the stomach
